# Migration and risk of schizophrenia and bipolar disorder: A Swedish national study

**DOI:** 10.1016/j.schres.2023.08.022

**Published:** 2023-09-02

**Authors:** Natassia Robinson, Alexander Ploner, Roxana Müller-Eberstein, Paul Lichtenstein, Kenneth S. Kendler, Sarah E. Bergen

**Affiliations:** a Department of Medical Epidemiology and Biostatistics, Karolinska Institutet, Stockholm, Sweden; b Virginia Institute for Psychiatric and Behavioral Genetics, Department of Psychiatry, Virginia Commonwealth University, Richmond, VA, United States of America

**Keywords:** Schizophrenia, Bipolar disorder, Migration, Ethnicity

## Abstract

**Objective:**

Prior studies report increased risk of schizophrenia (SCZ) in migrants relative to the native-born population; however, few have investigated bipolar disorder (BD) and migrant characteristics which may influence risk. We aimed to examine the risk of SCZ and BD in migrants and their children relative to those of Swedish ancestry, and whether risk varied by age at migration, region of origin, sex, and parental migrant status.

**Methods:**

We conducted a nested case-control study using 5539 SCZ cases and 20,577 BD cases diagnosed 1988–2013, individually matched to five population-based controls by birth year and sex. Conditional logistic regression was used to evaluate the risk of SCZ and BD by migrant status, region of origin and age at migration, with models stratified by sex.

**Results:**

First-generation migrants had increased risk of SCZ and decreased risk of BD. There was a distinct pattern of risk for SCZ by age at migration. Childhood migrants from all regions had increased risk of SCZ, particularly those from Africa. In contrast, risk for BD declined with age at migration, with increased risk only in Nordic child migrants. SCZ and BD diagnoses were decreased in adult migrants, elevated in second-generation migrants (with risk differing by number of migrant parents and greater for those with migrant fathers) and higher in male migrants (vs. female).

**Conclusions:**

Age at migration, sex, and region of origin affect risk of SCZ and BD. Further research is required to determine how migration-related factors influence disease etiology and the receipt of these diagnoses.

## 1. Introduction

In addition to having clinically overlapping features, schizophrenia (SCZ) and bipolar disorder (BD) have substantial genetic overlap (Lichtenstein et al., 2009; Bulik-Sullivan et al., 2015). However, the extent to which environmental risk factors are shared between the disorders is less known. Migration is a well-established environmental risk factor for SCZ (Henssler et al., 2020), yet findings are inconsistent across the few studies that have investigated migration and BD, possibly due population differences or variation in the definitions of the migrant characteristics and BD diagnoses examined (Dykxhoorn et al., 2019; Cantor-Graae and Pedersen, 2013). There is a predominance of studies on psychosis, a central feature of SCZ and present in about half of BD cases (Coryell et al., 2001; American Psychiatric Association, 2013), with meta-analyses reporting increased risk of psychosis in both first- and second-generation migrants (Cantor-Graae and Selten, 2005; Henssler et al., 2020; Bourque et al., 2011). There are fewer large-scale studies on the individual disorders, particularly for BD.

To identify high-risk groups and elucidate the underlying mechanisms, it is important to investigate characteristics of migrants potentially influencing risk, such as age, region of origin, and sex, and, for second-generation migrants, whether one or both parents were migrants. For SCZ, greatest risk has been observed for migration in early life, including infancy (Veling et al., 2011), childhood (Kirkbride et al., 2017), and early teenage years (Anderson et al., 2015). A Swedish study found greatest risk for BD without psychosis for those who migrated during infancy (0–2 years), and decreased risk at older ages (Dykxhoorn et al., 2019). However, not all studies support age-specific effects for SCZ (Cantor-Graae et al., 2003) or affective psychotic disorders (Dykxhoorn et al., 2019). Whether age effects differ by region of origin has not been investigated for migrants in Sweden.

Sex differences for these diagnoses are becoming increasingly recognized, whereby more females are diagnosed with BD and more males with SCZ (Aleman et al., 2003; Dell’Osso et al., 2021). Globally, the proportion of male and female migrants is similar, with some geographical variation, particularly for migrant workers (IOM, 2019); however, sex differences in the context of migration and risk of SCZ or BD have not been comprehensively examined.

Sweden has an increasingly diverse population with migrants comprising approximately 20 % of the population (IOM, 2019). This, along with universal healthcare and high-quality national register data, make it an ideal setting to study migration and psychiatric disorders. Here, we investigate the risk of SCZ and BD diagnoses in first- and second-generation migrants compared with those of Swedish ancestry. Although region of origin and age at migration have both separately been shown to influence risk, to date, no study has simultaneously investigated these factors. Building on earlier research, we concurrently examine the role of age at migration and region of origin, parental migrant status, and evaluate sex differences in risk of SCZ and BD.

## 2. Methods

### 2.1. Study design and population

**Fig. 1.**
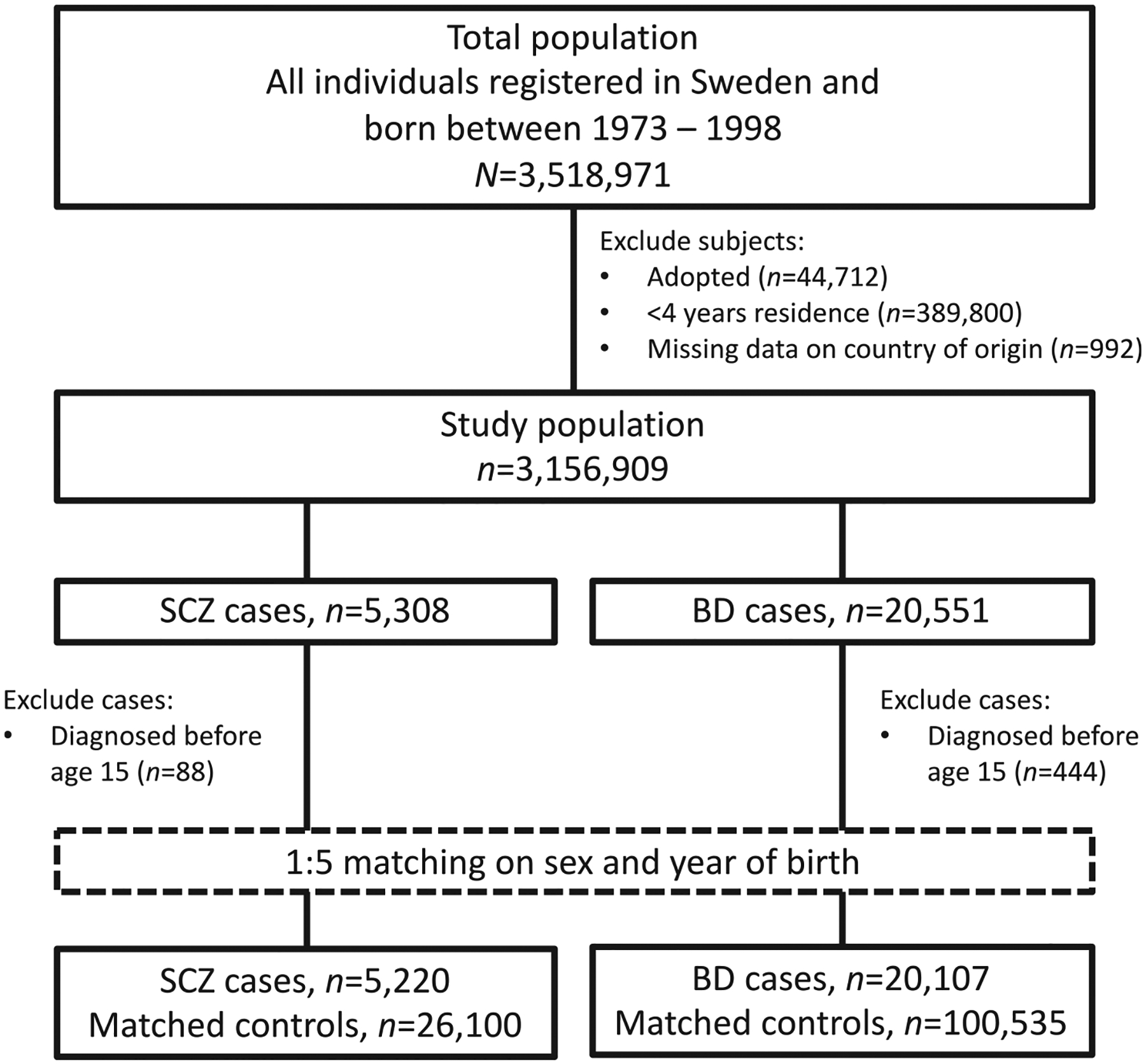
Flow chart of study population selection.

We conducted a register-based, population-wide nested case-control study among persons born between 1973 and 1998 and resident in Sweden at some point during the study period, 1988–2013. Members of this source population were identified via the Swedish Total Population Register (TPR) (Fig. 1).

All cases of SCZ and BD in the source population were identified at their first diagnosis recorded in the National Patient Register (NPR). In addition to the precise clinical definitions described below under Outcomes, cases included in the study were required to (i) be at least 15 years old at diagnosis, (ii) have no previous history of emigration from Sweden in the Migration Register, and (iii) have been resident in Sweden at least four years before first diagnosis. The chosen four-year threshold represents the minimum required time for permanent residency which was introduced to omit short-term migrants like international students, to allow for a period of acclimatization to the healthcare system and culture for migrant subjects, and in the absence of migrant diagnostic history, filters out those with prior diagnoses. Adopted individuals were excluded. For each case included in the study, we identified five controls from the TPR who were matched on age and sex, fulfilled the same residency requirements, and had no diagnosis of SCZ or BD, respectively, in the NPR at the date of diagnosis of their matched index case (incidence density sampling). Register linkages were based on the unique personal identification number assigned to Swedish residents for administrative purposes. This study was approved by the Regional Ethics Review Board in Stockholm, Sweden (DNR 2013/862–31/5), waiving informed consent due to use of retrospective population-based registers with anonymized identifiers.

### 2.2. Outcomes

We defined diagnoses in the National Patient Register with *International Classification of Diseases, Ninth Revision* (ICD-9, 1987–1996), and *International Statistical Classification of Diseases and Related Health Problems, Tenth Revision* (ICD-10, 1997–present) codes for SCZ (295 and F20, excluding simple, acute, latent and schizoaffective types) and BD (296 and F30–31, excluding unipolar melancholic, other and unspecified types) (supplementary methods). We identified inpatient (complete since 1987) and outpatient specialist care (since 2001) diagnoses in the NPR between 1 January 1988 and 31 December 2013. Both disorders were diagnosed using a non-hierarchical diagnostic structure, meaning an individual could contribute to analyses of both SCZ and BD, if they had received both diagnoses during the study time period.

BD was further defined by the following clinical diagnoses: ICD-9 with prior exclusions, and ICD-10 with psychotic symptoms, without psychotic symptoms, or unspecified (see supplementary methods for details). The subset of diagnoses specifying with/without psychotic symptoms were too few to examine as outcomes but are reported in the descriptive statistics (Table S2).

### 2.3. Exposures

Migrant status was defined using the TPR for first-generation migrants, and through identification of migrant parents for second-generation migrants using the Multigeneration Register. Participants were classified as (i) Swedish-ancestry when born in Sweden to two Swedish-born parents; (ii) first-generation migrants when born outside of Sweden, and; (iii) second-generation migrants when born in Sweden with one or both parents born outside of Sweden. Increased risk in second-generation migrants implicates other factors beyond the direct stress of migration and resettling. Due to potentially different mechanisms operating across generations, it is important to study these groups separately from first-generation migrants. For second-generation migrants, if there were no parental birth region data, parental ancestry could not be assumed and they were classified as missing (BD matched sample, n = 704, 0.6 %, SCZ matched sample, n = 185, 0.6 %). First-generation migrants were further categorized by region of origin and age at migration, and second-generation migrants by maternal/paternal migrant status and number of migrant parents.

The regions reported by Statistics Sweden were Sweden, Nordic, European Union (28 countries, EU28), Europe (non-EU28), Africa, North America, South America, Asia, Soviet Union and Oceania (Supplementary methods). Soviet Union was merged with Europe non-EU28 for analysis, and migrants missing region of origin were excluded. There were few individuals from Oceania in the matched samples (SCZ data, n = 34 (0.1 %); BD data, n = 75 (0.1 %)); thus, this group was excluded from regional analyses.

Age at migration was analysed as a single continuous exposure and also incorporated into the regional analysis as a categorical exposure (child <18 years; adult ≥18 years). Time to diagnosis in migrant cases was defined as the difference between the date of immigration and first diagnosis.

### 2.4. Statistical analysis

We generated descriptive statistics of the sample by case-control and migrant status. Conditional logistic regression was used to calculate odds ratios, which (in combination with incidence density sampling) can be interpreted as incidence rate ratios (IRR), for risk of SCZ and BD in relation to migrant status, region, age at migration, region-by-age, and parental migrant status. IRR with 95 % confidence intervals (CI) are presented, with all estimates relative to those of Swedish ancestry. We investigated nonlinear associations for age at migration using natural spline functions, and explored a range of degrees of freedom and selected the best fitting model by visual comparison and likelihood ratio tests. All analyses were stratified by sex with two-sided Wald tests to evaluate sex differences. We conducted sensitivity analyses (including rematching) without the residency requirement of four years to examine whether this impacted the patterns of risk by age at migration.

Statistical analyses were conducted in R (version 4.0.5), using the Survival package (version 3.2–10) for regression analyses (Therneau and Lumley, 2014).

## 3. Results

### 3.1. Descriptive characteristics

We identified 5220 SCZ cases and 20,107 BD cases during the study period (Table 1). First-generation migrants comprised 20 % of SCZ cases, and only 7 % of BD cases. Cases in second-generation migrants were more comparable (21 % SCZ, 18 % BD). There was regional variation with proportionally more SCZ cases in migrants from Africa and Europe (non-EU28), and few from Europe, and BD cases in migrants from the Nordic and American regions (Table 1).

Most SCZ cases were male (68 %) and BD cases female (66 %) (Table 1). Across regions, the sex distribution in cases was similar, except for Africa and South America, which had higher proportions of male SCZ cases (77 %), and for Europe (EU28) and North America which had fewer female BD cases (63 %) (Tables S1 and S2).

Median age at diagnosis was similar for both disorders (25–26 years) (Table 1). Swedish-born individuals were diagnosed at an earlier age than first-generation migrants (Table S1). Following arrival in Sweden, the time to first diagnosis for migrants was shorter for SCZ than for BD (median SCZ 13 years; BD 15 years) (Table 1).

It was more common for migrants to have received a diagnosis of both SCZ and BD (than BD only): for example, this occurred in 11 % of African and 8 % of European (non-EU28) cases, compared to just 2 % of Swedish ancestry cases (Table S2). Similarly, BD with psychosis accounted for 19 % of cases in those from Africa and 11 % from Europe (non-EU28), but only 5 % of cases with Swedish ancestry.

### 3.2. Study findings

In the matched analysis, in first-generation migrants, risk of SCZ was increased (IRR 1.28, 95 % CI 1.19–1.39) but risk of BD was decreased (IRR 0.40, 95 % CI 0.37–0.42), compared to those of Swedish ancestry (Tables S3–S4). For both SCZ and BD, female first-generation migrants had significantly lower risk than males.

### 3.3. Effect modification by age at migration

**Fig. 2.**
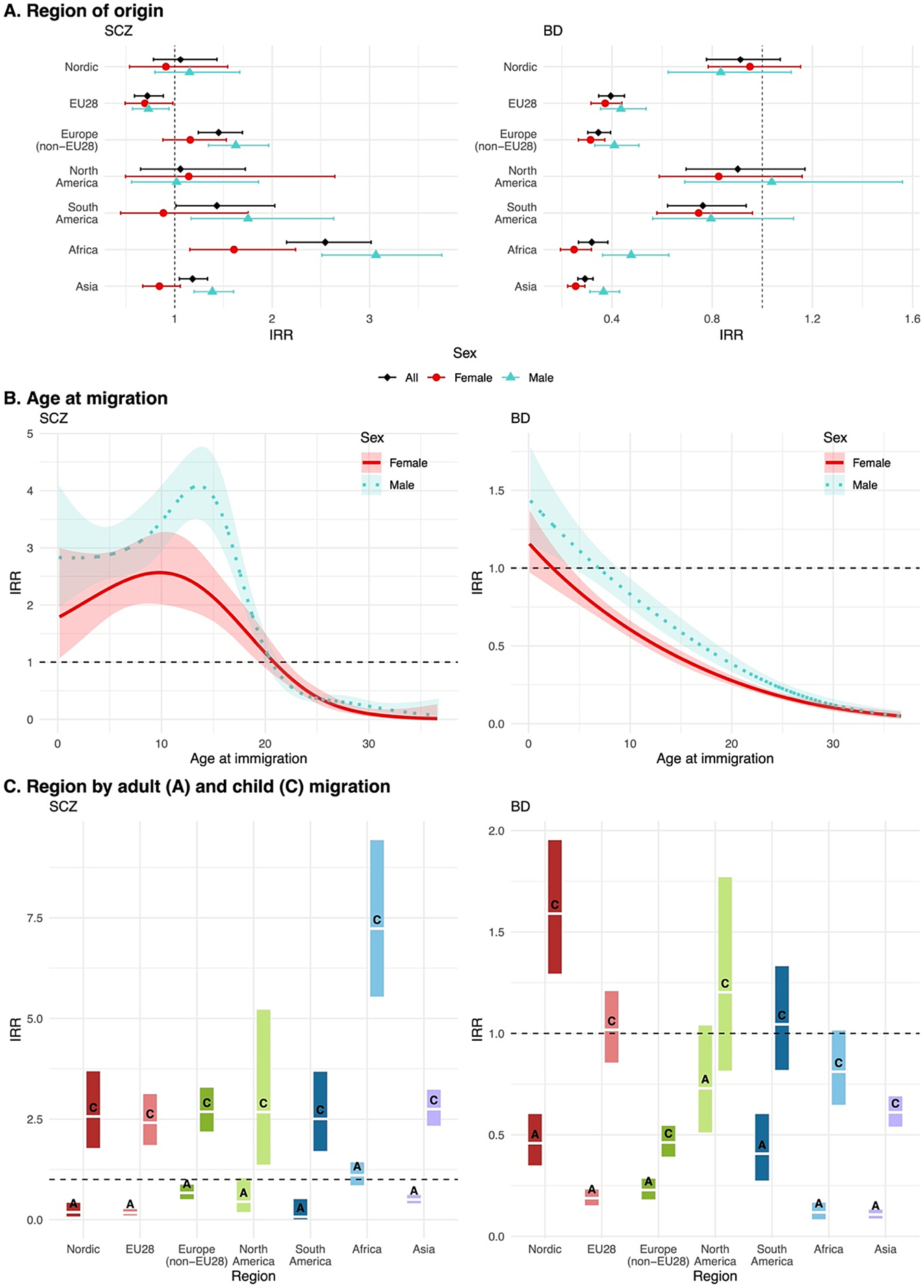
IRR for SCZ and BD by migration exposures. (A) Disease risk by region and sex; (B) disease risk as smooth function (spline) of age at migration; and (C) disease risk by region and migration in childhood (C, <18 years) or adulthood (A, ≥18 years). The reference group is Swedish ancestry. Points or solid lines represent IRR, and bars or shaded areas the corresponding 95 % confidence intervals.

Age at migration had a considerable influence on both disorders, but with clear differences in how it shaped risk. For SCZ, the risk gradually increased in early childhood, peaked between ages 10–15 years and declined steeply thereafter (Fig. 2B). SCZ risk was lower for female migrants and peaked at earlier ages of migration (age 10, IRR 2.5, 95%CI 2.0–3.5) than for male migrants (age 14, IRR 4.0, 95%CI 3.5–4.8). Those migrating after age 20 had lower rates of SCZ than those of Swedish ancestry. Conversely, BD risk decreased with age at migration, with marginally (males) or non-elevated (females) risk for very young migrants (0–4 years), and smoothly declining risk with increasing age at migration. In the sensitivity analyses without the stringent four years residency requirement, there were an additional 319 SCZ and 470 BD first-generation migrant cases. The patterns of risk were strikingly similar with mostly overlapping confidence intervals for both SCZ and BD and minor divergence at older ages of migration (Supplementary Fig. 1). Peak risk around (age 10–11) for SCZ was slightly lower when employing a buffer period.

### 3.4. Region by age effects

Despite regional variation in SCZ risk in the unadjusted analysis (Fig. 2A), the IRR across most regions were comparable after controlling for age at migration (Fig. 2C). Risk of SCZ was consistently elevated across all regions for those who migrated in childhood (IRR 2.41–7.23), compared to those of Swedish ancestry (Fig. 2C, Table S3). In contrast, those who migrated in adulthood had decreased risk. The exception was migrants from Africa: those who migrated in childhood had considerably higher risk of SCZ (IRR 7.23, 95 % CI 5.54–9.43), especially males (IRR males 8.39 vs females 4.33; Wald test p < 0.05); and those who migrated as adults had slightly, but not significantly, increased risk versus Swedish ancestry (IRR 1.11, 95 % CI 0.86–1.43) (Table S3).

Stratifying the regions by age at migration revealed elevated risk of BD for childhood migrants from Nordic countries (IRR 1.59, 95 % CI 1.29–1.95) (Fig. 2C, Table S4). Those who migrated in childhood from Europe (non-EU28), Africa, and Asia had decreased risk of BD, whilst risk in those from EU28, North America, and South America did not differ significantly from Swedish ancestry. Adult migrants from all regions had lower risk of BD than individuals of Swedish ancestry.

Risk of both disorders was generally greater in male migrants, with statistically significant sex differences for childhood migrants from Africa and Asia (Wald-test p < 0.05, Tables S3–S4).

### 3.5. Variable risk in second-generation migrants

**Fig. 3.**
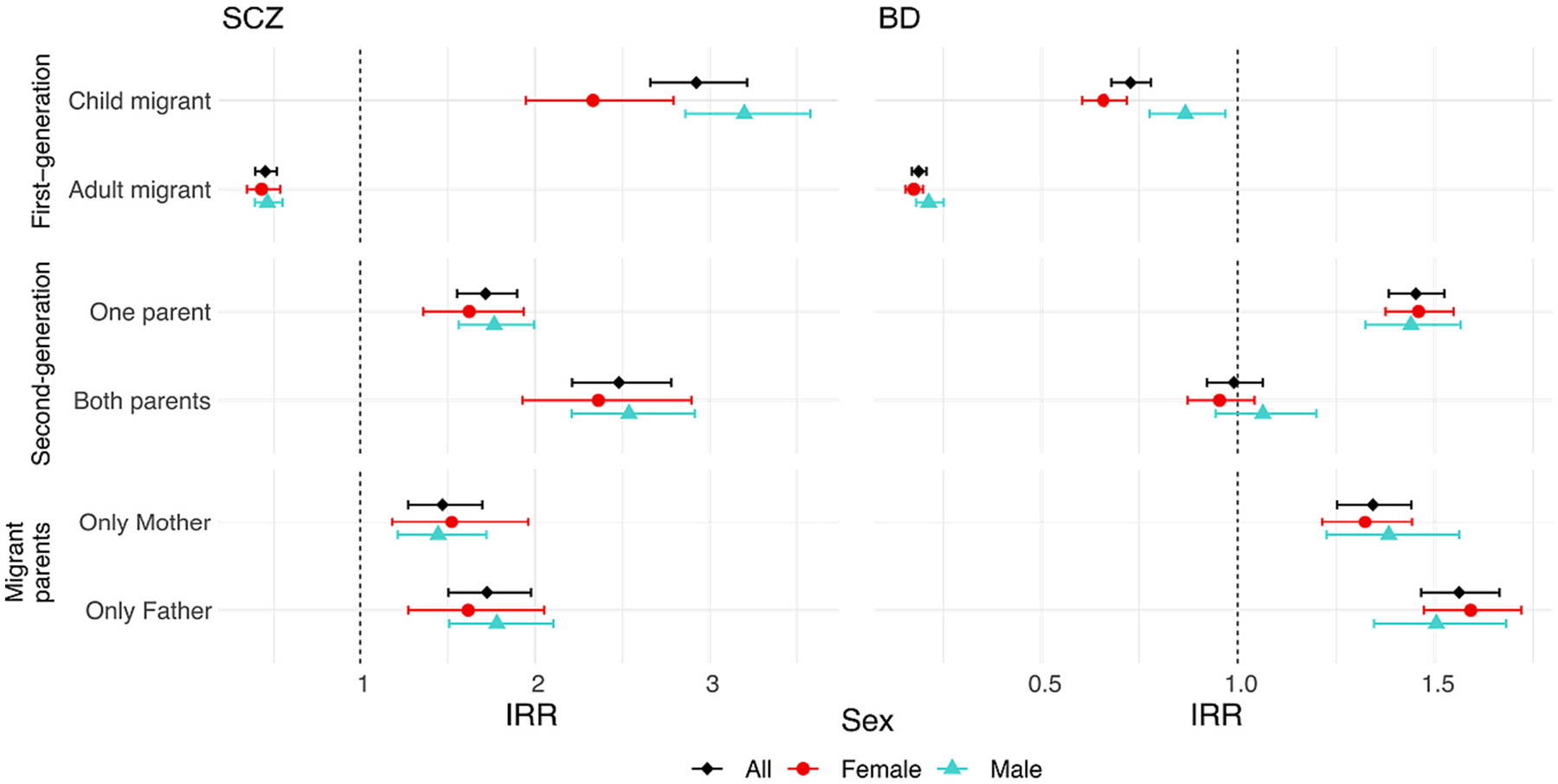
IRR for SCZ and BD stratified by migration in childhood (<18 years) or adulthood (≥18 years) for first-generation migrants, and by maternal/paternal migrant parents and number of migrant parents for second-generation migrants. The reference group is Swedish ancestry. Bars denote 95 % confidence intervals.

Among subjects raised in Sweden, we found a risk gradient for SCZ, going from lowest risk for those closest to Swedish ancestry (one migrant parent, one Swedish-born parent), to intermediate risk in those born in Sweden to two migrant parents, and highest risk in those born abroad who migrated in childhood (Fig. 3). For BD, we found an inverted gradient across the same exposure groups, where individuals with one migrant parent were the only group with increased risk of BD (IRR 1.45, 95 % CI 1.38–1.52) (Table S4). Risk for both disorders was slightly greater for those with a migrant father vs mother and this difference was significant for BD (Wald test p = 0.002). We only observed strong sex differences in risk for either disorder in child migrants (p values < 0.01, Tables S3–S4).

## 4. Discussion

### 4.1. Overall findings

In first-generation migrants, analysis of age patterns of migration revealed that SCZ risk differed markedly, with increased risk for child migrants and decreased risk for adult migrants. In contrast, risk for BD was consistently lower in first-generation migrants, with a smooth decline in risk with increasing age at migration.

Investigating region together with age at migration revealed novel findings. The excess risk of SCZ among child migrants was consistent across regions of origin, except for migrants from Africa, whose excess risk was more than double that of children from other regions. Risk of BD among childhood migrants varied between regions of origin, but only child migrants from the Nordic region had significantly higher risk than those of Swedish ancestry. Male migrants generally had higher risk for both disorders across all ages and regions.

Second-generation migrants also had elevated risk of SCZ and BD, but the risk patterns varied based on the number of migrant parents. Individuals with one migrant and one Swedish-born parent had a higher risk of BD, whilst number of migrant parents was associated with incremental increases in risk of SCZ. Moreover, having a migrant father was associated with a slightly higher risk for both disorders than having a migrant mother.

### 4.2. Comparisons with previous literature

Our findings of elevated risk of SCZ in first- and second-generation migrants are in agreement with previous studies in European countries (Cantor-Graae and Selten, 2005), including Sweden (Dykxhoorn et al., 2019). We were able to perform more fine-tuned analysis of age at migration than in prior studies, and our central finding was that age at migration was strongly associated with SCZ risk, with distinct sex differences in peak risk timing and magnitude. Without age adjustment, the comparatively low risk in adult migrants obscured the elevated risk in child migrants from *all* regions. Elevated risk of SCZ in people who migrate before age 18 is consistent with findings for psychotic disorders (Anderson and Edwards, 2020), and with studies in the UK and Canada which find peak risk of psychosis for those migrating in late childhood/early adolescence (Kirkbride et al., 2017; Anderson et al., 2015). In contrast, age at migration was strongly, inversely associated with risk of BD. Few large studies have investigated age at migration and BD, but a previous Swedish study also reported progressively decreasing risk of BD at older ages of migration (Dykxhoorn et al., 2019).

We extend the study by Dykxhoorn et al., with novel findings based on region, age at migration, a larger sample size, and a longer study period. They found all age groups had elevated risk of SCZ/schizo-affective diagnoses, and in contrast to our results, highest risk in young adults migrating at ages 19–29 (HR = 2.77). However, applying a two-year buffer period decreased the number of young adult cases by 63 % (n = 96 to n = 36) and reduced the estimate from 2.77 (95%CI 1.64–4.69) to a non-significant increase of 1.81 (95%CI 0.80–3.71), implying that this age group in particular has a higher likelihood of prevalent cases or acute onset. Distinctions from our study also include differences in diagnostic coding (we focus on SCZ only) and they examine a later study period (1982–2011), which may also lead to dissimilar findings. We also found that using a buffer period in our study led to more conservative estimates. Applying a buffer period in migration studies likely provides more reliable estimates and more balanced comparison with the native population by accounting for potential barriers to receiving a diagnosis such as familiarity with healthcare systems and language proficiency, and omitting short-term migrants who are less likely to receive a diagnosis and may dilute the effects.

There are two key theories about the relationship between migration and psychiatric disorders: the “healthy migrant” hypothesis, which suggests that healthier, better-educated people with lower rates of mental illness are more likely to migrate (Rubalcava et al., 2008), and the stress hypothesis which suggests that migration and the accompanying experiences cause distress and greater risk of psychopathology among migrants (Bhugra and Jones, 2001). For those who migrate voluntarily (i.e., not refugees), it is plausible that both mechanisms apply, corresponding to our observations of protective effects in “healthy” adult migrants, and detrimental effects for children migrating during a sensitive developmental period. Nevertheless, increased rates of SCZ and BD in second-generation migrants indicate that post-migration factors persist beyond first-generation migrants.

The risk of SCZ was consistently higher for childhood migrants from all regions, suggesting that the experience of relocating during this developmental period has a detrimental impact regardless of origin; whereas BD risk was variable across regions of origin. However, risk of SCZ was greatest for those migrating in childhood from Africa. These findings add to a growing body of evidence that migrants of African ancestry in European countries have considerably higher risk of SCZ (Cantor-Graae and Selten, 2005), consistent with stress-related adversities such as discrimination and/or social exclusion (Bourque et al., 2011). The impact may be greater in the childhood-adolescent period, as migrants from sub-Saharan Africa in the Netherlands have much higher rates of treatment for psychosis evident as early as the first year of arrival, for all ages at migration examined but particularly age 10–20 years (Termorshuizen and Selten, 2022).

Additionally, in our sample, proportionally more migrants from Africa had a diagnosis of both SCZ and BD (versus only BD) compared to Swedish ancestry and other migrants, suggesting higher rates of misdiagnosis or different diagnostic journeys. Studies from the United States and the United Kingdom have also found that black patients receive more severe SCZ spectrum disorder diagnoses over BD diagnoses compared to white patients (Haeri et al., 2011). We found that compared to BD cases with Swedish ancestry, migrant cases from Africa were more likely to be male or have a diagnosis of BD with psychosis. There are several possible explanations, for example: cultural variation in clinical presentation; cultural reluctance or barriers to accessing psychiatric services and concomitant delays in treatment, leading to more severe symptomology; population differences in disease risk; or clinicians perceiving symptoms as more severe than in those of Swedish ancestry. If cultural factors negatively influence diagnosis of BD more than SCZ, that may explain why we only identified higher rates of BD in Nordic childhood migrants (the region most comparable to Sweden), and in second-generation migrants with one Swedish-born and one migrant parent.

Higher rates of BD in those with one migrant parent and higher rates of SCZ for two migrant parents have also been observed previously in a Danish study (Cantor-Graae and Pedersen, 2013). Our study had more cases and greater power, which is perhaps why we also identified parental sex differences, with higher risk in children born in Sweden with migrant fathers. As male migrants generally fared worse across all exposures, this could indicate that there are different obstacles faced by male migrants.

### 4.3. Strengths & limitations

The Swedish registers are nationally representative, with high-quality, comprehensive data and good diagnostic validity (Ekholm et al., 2005; Sellgren et al., 2011; Ludvigsson et al., 2011). In our long follow-up period, we identified numerous SCZ and BD cases, and to our knowledge, this is the largest study of migration and BD to date. Despite our large sample, we had limited statistical power for some subgroup analyses, such as migrants from North and South America, in the age-by-region analysis. Therefore, even larger sample sizes will be required to further investigate regional differences in early-life migration and SCZ risk.

Sweden has a diverse international population with heterogeneous regions of origin. National statistics of immigration into Sweden show fluctuations in migrant populations during our study period, with an influx from Chile (South America) in the 1970–80s; former Yugoslavia (non-EU28) in the 1990s; and Iran (Asia), Iraq (Asia) and Somalia (Africa) in the 2000s (SCB, 2021). The largest foreign-born population residing in Sweden originates from Finland (Nordic region, n ≅ 160,000 in 2013) (SCB, 2021). Although we cannot discount the possibility of differences by country of origin within regions, individual countries constitute a relatively small proportion of the estimated 1.6 million foreign-born individuals in Sweden (2013) and are therefore unlikely to dominate the estimates we observed. Reasons for migration, whether voluntary (e.g. labour) or involuntary (e.g. refugees), vary both by region and within regions. Although we could not differentiate in our data, labour migration is more common in those with EU-citizenship, and family reunification and asylum for non-EU citizens (SCB, 2021).

The matched design controlled for age, period effects and sex. Socioeconomic status, however, requires cautious interpretation regarding psychiatric disorders, as downward social mobility could be a consequence of symptoms and not necessarily a confounder of the exposure-outcome relationship. Socioeconomic data is disproportionately missing for migrants, but previous migration studies using Swedish register data report that estimates were somewhat attenuated but not explained by income (Dykxhoorn et al., 2019).

Although healthcare is subsidized and accessible for all residents, health system literacy is likely higher in native-born citizens. Migrants in Sweden generally have lower use of psychiatric care during the first decade, particularly for specialist outpatient services, with utilization increasing over time (Hollander et al., 2020). To mitigate this, we required migrants be resident for at least four years, but less severe cases may still be under-detected. In addition to having accessible health care, Sweden is recognized as having high standards for working conditions, gender equality, and programs facilitating integration of migrants, such as freely available language and social orientation (Swedish for Immigrants) courses (University of Essex Human Rights Centre Clinic, 2013; Rechel et al., 2013); thus, outcomes for migrants may be more favourable than in other settings.

## 5. Conclusions

This study supports migration in childhood as a risk factor for SCZ and to a lesser extent BD and offers new evidence regarding variation by sex, age at migration, and region of origin. The difference in risk according to age at migration highlights the necessity of incorporating age in future studies of migration. Studies investigating the specific experiences of male migrants, and designs that can distinguish whether childhood is a sensitive period or if accumulated years of residence impacts risk may reveal more about the underlying mechanisms. The precise mechanisms underlying the substantially higher risk of SCZ in childhood migrants from Africa, and higher rates of both SCZ and BD diagnoses warrant further investigation, along with the potential effects of diagnostic bias on differential diagnoses. Given the harmful implications of misdiagnosis on treatment, possible disparities need to be acknowledged and addressed, as well as the cross-cultural validity of diagnostic criteria (Alarcón et al., 2002).

Although Sweden records no ancestry, ethnicity, or cultural data, there are marked health inequalities in foreign-born individuals (University of Essex Human Rights Centre Clinic, 2013), and the extent to which systemic racism and unconscious bias may contribute to these findings is unknown (Bradby et al., 2019). Concomitantly, there may be true regional variation in SCZ and BD prevalence or genetic risk, but this remains uncertain in the absence of high-quality global epidemiological and genetic data. In general, greater diversity in migration studies is required, as most have been conducted in countries with majority European-ancestry populations.

Despite a high degree of shared genetic risk between BD and SCZ, we find evidence that migration is a distinguishing environmental risk factor for these diagnoses. With increasing global migration, future studies need to establish how migration-related factors influence disease etiology or diagnostic practices.

## Supplementary Material

Supplement

## Figures and Tables

**Table 1:** Demographic characteristics of SCZ/BD cases and sex- and age-matched controls.

Characteristic	SCZ		BD	
	Control, N = 26,100	Case, N = 5220	Control, N = 100,535	Case, N = 20,107
Migrant status				
Swedish ancestry	18,048 (69.5 %)	3057 (59.2 %)	70,484 (70.5 %)	15,029 (75.4 %)
First-generation	4769 (18.4 %)	1036 (20.1 %)	16,703 (16.7 %)	1426 (7.2 %)
Second-generation	3155 (12.1 %)	1070 (20.7 %)	12,809 (12.8 %)	3487 (17.5 %)
Sex				
Male	17,625 (67.5 %)	3525 (67.5 %)	34,325 (34.1 %)	6865 (34.1 %)
Female	8475 (32.5 %)	1695 (32.5 %)	66,210 (65.9 %)	13,242 (65.9 %)
Age at migration (years)	23 (14–27)	13 (8–18)	21 (12–26)	11 (6–20)
Region of origin				
Sweden	21,331 (81.7 %)	4184 (80.2 %)	83,832 (83.4 %)	18,681 (92.9 %)
Nordic	286 (1.1 %)	53 (1.0 %)	949 (0.9 %)	190 (0.9 %)
EU28	925 (3.5 %)	111 (2.1 %)	3057 (3.0 %)	263 (1.3 %)
Europe (non-EU28)	892 (3.4 %)	224 (4.3 %)	3298 (3.3 %)	248 (1.2 %)
North America	114 (0.4 %)	20 (0.4 %)	364 (0.4 %)	71 (0.4 %)
South America	185 (0.7 %)	43 (0.8 %)	694 (0.7 %)	113 (0.6 %)
Africa	530 (2.0 %)	221 (4.2 %)	1859 (1.8 %)	128 (0.6 %)
Asia	1805 (6.9 %)	362 (6.9 %)	6416 (6.4 %)	404 (2.0 %)
Oceania	32 (0.1 %)	2 (0.0 %)	66 (0.1 %)	9 (0.0 %)
Age at diagnosis (years)		25.3 (21.8–29.6)		25.7 (21.3–30.8)
Time to diagnosis (migrants, years)		13 (9–18)		15 (9–21)

Values are n (column %) or Median (IQR). Migrant status excludes Swedish-born individuals with missing parental ancestry data (SCZ, n = 185, 0.6 %; BD, n = 704, 0.6 %).
