# Supplementary material for: Migration and risk of schizophrenia and bipolar disorder: A Swedish national study
Source: Schizophr Res. Author manuscript; Available in PMC 2024 Jul 23. (PMC11265771; doi:10.1016/j.schres.2023.08.022)
Supplement: Supplement [file NIHMS2009738-supplement-Supplement.docx]

### Supplementary methods

**ICD diagnostic codes**

*International Classification of Diseases, Ninth Revision* (ICD-9, 1987-1996), and *International Statistical Classification of Diseases and Related Health Problems, Tenth Revision* (ICD-10, 1997-present) codes were used for defining outcomes.

SCZ was defined as ICD9 295 and ICD10 F20 with latent (ICD9 295F), simple (ICD9 295A; ICD10 F20.6), acute (ICD9 295E), post-schizophrenic depression (ICD10 F20.4), and schizoaffective disorders (ICD9 295H) excluded.

BD was defined as ICD9 296 with unipolar aﬀective psychosis, melancholic form (ICD9 296B), other (ICD9 296W) and unspecified (ICD9 296X) excluded, and all ICD 10 F30 and F31.

BD diagnoses were subclassified as: ICD-9 (296, excluding 296B, 296W, 296X), ICD10 specified with psychotic symptoms (F30.2, F31.2, F31.5), specified without psychotic symptoms (F30.1, F31.1, F31.4), or other/non-specified ICD-10 diagnoses (F30.0, F30.8, F30.9, F31, F31.0, F31.3, F31.6, F31,8, F31.9).

**Regional groupings provided by Statistics Sweden**

| Africa | Algeria, Angola, The Arab Republic Of Egypt, Benin, Botswana, Burkina Faso, Burundi, Central African Republic, The Comoros, Djibouti, Egypt, Equatorial Guinea, Ivory Coast, Eritrea, Ethiopia, French Morocco, Gabon, Gambia, Ghana, Guinea, Guinea-Bissau, Cameroon, Cape Verde, Kenya, Congo, Congo, Democratic Republic, Lesotho, Liberia, Libya, Madagascar, Malawi, Mali, Morocco, Mauritania, Mauritius, Mozambique, Namibia, Niger, Nigeria, Rwanda, Sao Tome And Principe, Senegal, Seychelles, Sierra Leone, Somalia, Sudan, Swaziland, South Africa, South Sudan, Tanzania, Tchad, Togo, Tunisia, Uganda, Zambia, Zanzibar |
| --- | --- |
| Asia | Afghanistan, The Arab Emirates, United States, Armenia, Azerbaijan, Bahrain, Bangladesh, Bhutan, Brunei Darussalam, Philippines, Gaza Area, Georgia, Hong Kong, India, Indonesia, Iraq, Iran, Israel, Japanese, Jordan, Cambodia, Kazakhstan, China, Kyrgyzstan, Korea, North-, Korea, South-, Kuwait, Laos, Lebanon, Malay League, Malaysia, Maldives, Mongolia, Myanmar, Nepal, Oman, Pakistan, Palestine, Qatar, Saudi Arabia, Sikkim, Singapore, Sri Lanka, Sydjemen, Syria, Tajikistan, Taiwan, Thailand, Turkmenistan, Uzbekistan, Vietnam, Vietnam, Rope, West Bank, Yemen, East Timor |
| Europe (EU28) | Austria, Belgium, Bulgaria, Cyprus, Danzig, Estonia, France, Greece, Ireland, Italy, Croatia, Latvia, Lithuania, Luxembourg, Malta, Netherlands, Poland, Portugal, Romania, Slovakia, Slovenia, Spain, Great Britain And N. Ireland, Czech Republic, Czechoslovakia, German Democratic Republic (DDR), Germany, Hungary |
| Europe (non-EU28) | Albania, Andorra, Bosnia Herzegovina, Gibraltar, Yugoslavia, Kosovo, Liechtenstein, Macedonia, Moldova, Monaco, Montenegro, Russia, San Marino, Switzerland, Serbia, Serbia And Montenegro, Turkey, Ukraine, Vatican City, Belarus |
| North America | Anguilla, Antigua And Barbuda, Bahamas, Barbados, Belize, Bermuda, Costa Rica, Dominica, Dominican Republic, El Salvador, Grenada, Guatemala, Haiti, Honduras, Jamaica, The Virgin Islands, British, Canada, Cuba, Mexico, Nicaragua, Panama, St Kitts And Nevis, St Lucia, St Vincent And The Grenadines, Trinidad And Tobago, USA |
| South America | Argentina, Bolivia, Brazil, Chile, Colombia, Ecuador, Guyana, Paraguay, Peru, Suriname, Uruguay, Venezuela |
| Oceania | Australia, Fiji, Kiribati, Marshall Islands, Micronesia, Nauru, New Zealand, Palau, Papua New Guinea, Solomon Islands, Samoa, Tonga, Tuvalu, Vanuatu, Western Samoa |
| Sweden | Sweden |
| Nordic | Denmark, Finland, Iceland, Norway |
| The Soviet Union | The Soviet Union |

### Supplementary TABLES

Table S1 Demographic and clinical characteristics of SCZ sample by migrant status and region of origin

|  |  | Migrant status | | |  | Region of origin (first-generation migrants) | | | | | | | |  |
| --- | --- | --- | --- | --- | --- | --- | --- | --- | --- | --- | --- | --- | --- | --- |
| Characteristic | Swedish ancestry  N = 21,105 | First-generation  N = 5,805 | Second-generation  N = 4,225 | p-value^1^ |  | Nordic  N = 339 | EU28  N = 1,036 | EUR (non-EU28)  N = 1,116 | N. America  N = 134 | S. America  N = 228 | Africa  N = 751 | Asia  N = 2,167 | Oceania  N = 34 | p-value^1^ |
| **All subjects** |  |  |  |  |  |  |  |  |  |  |  |  |  |  |
| **Sex** |  |  |  | 0.070 |  |  |  |  |  |  |  |  |  | 0.9 |
| *Male* | 14,259 (67.6%) | 3,861 (66.5%) | 2,902 (68.7%) |  |  | 220 (64.9%) | 679 (65.5%) | 741 (66.4%) | 95 (70.9%) | 154 (67.5%) | 507 (67.5%) | 1,435 (66.2%) | 30 (88.2%) |  |
| *Female* | 6,846 (32.4%) | 1,944 (33.5%) | 1,323 (31.3%) |  |  | 119 (35.1%) | 357 (34.5%) | 375 (33.6%) | 39 (29.1%) | 74 (32.5%) | 244 (32.5%) | 732 (33.8%) | 4 (11.8%) |  |
| **Age at migration (years)** | - | 21 (12, 27) | - |  |  | 19 (4, 26) | 24 (14, 28) | 18 (11, 24) | 22 (12, 27) | 12 (5, 24) | 22 (14, 28) | 22 (13, 27) | 26 (23, 28) | **<0.001** |
| **Case status** |  |  |  | **<0.001** |  |  |  |  |  |  |  |  |  | **<0.001** |
| *Control* | 18,048 (85.5%) | 4,769 (82.2%) | 3,155 (74.7%) |  |  | 286 (84.4%) | 925 (89.3%) | 892 (79.9%) | 114 (85.1%) | 185 (81.1%) | 530 (70.6%) | 1,805 (83.3%) | 32 (94.1%) |  |
| *Case* | 3,057 (14.5%) | 1,036 (17.8%) | 1,070 (25.3%) |  |  | 53 (15.6%) | 111 (10.7%) | 224 (20.1%) | 20 (14.9%) | 43 (18.9%) | 221 (29.4%) | 362 (16.7%) | 2 (5.9%) |  |
| **Cases only** |  |  |  |  |  |  |  |  |  |  |  |  |  |  |
| **Sex (% Male)** | 2007 (65.7%) | 745 (71.9%) | 732 (68.4%) | **<0.001** |  | 36 (67.9%) | 72 (64.9%) | 157 (70.1%) | 13 (65.0%) | 33 (76.7%) | 170 (76.9%) | 262 (72.4%) | 2 (100.0%) | 0.3 |
| **Age at diagnosis (years)** | 25.2  (21.4, 29.2) | 26.6  (23.2, 30.9) | 24.6  (21.6, 29.0) | **<0.001** |  | 25.9  (21.9, 29.0) | 26.2  (22.9, 30.9) | 26.4  (23.0, 30.1) | 28.5  (25.2, 33.6) | 27.0  (22.7, 29.5) | 27.1  (23.4, 31.3) | 26.8  (23.2, 31.1) | 31.7  (27.8, 35.5) | 0.3 |
| **Diagnosis of SCZ & BD** | |  |  | **<0.001** |  |  |  |  |  |  |  |  |  |  |
| *SCZ diagnosis only* | 2,721 (89.0%) | 973 (93.9%) | 987 (92.2%) |  |  | 47 (88.7%) | 107 (96.4%) | 205 (91.5%) | 18 (90.0%) | 41 (95.3%) | 207 (93.7%) | 346 (95.6%) | 2 (100.0%) | NA |
| *SCZ & BD diagnoses* | 336 (11.0%) | 63 (6.1%) | 83 (7.8%) |  |  | 6 (11.3%) | 4 (3.6%) | 19 (8.5%) | 2 (10.0%) | 2 (4.7%) | 14 (6.3%) | 16 (4.4%) | 0 (0.0%) |  |
| **Time to diagnosis (migrants, years)** | - | 13 (9, 18) | - | - |  | 19 (13, 26) | 16 (11, 20) | 13 (9, 17) | 16 (11, 20) | 20 (15, 24) | 12 (7, 16) | 13 (8, 18) | 13 (10, 16) | **<0.001** |

n (column %); Median (IQR). ^1^ Kruskal-Wallis rank sum test for continuous variables, Pearson's Chi-squared test for categorical variables with all expected cell counts >=55. Oceania region excluded from statistical tests. NA, no statistical test performed due to few observations. Bold indicates p<0.05. Migrant status excludes Swedish-born individuals with missing parental ancestry data (n=185).

Table S2 Demographic and clinical characteristics of BD sample by migrant status and region of origin

|  | Migrant status | | | | |  | |  | | | Region of origin (first-generation migrants) | | | | | |  | | |
| --- | --- | --- | --- | --- | --- | --- | --- | --- | --- | --- | --- | --- | --- | --- | --- | --- | --- | --- | --- |
| **Characteristic** | Swedish ancestry  N = 85,513 | First-generation  N = 18,129 | Second-generation  N = 16,296 | p-value^1^ |  | | Nordic  N = 1,139 | | EU28  N = 3,320 | EUR (non-EU28)  N = 3,546 | | N. America  N = 435 | S. America  N = 807 | Africa  N = 1,987 | Asia  N = 6,820 | Oceania  N = 75 | | **p-value^1^** |  |
| **All subjects** |  |  |  |  |  |  |  | |  |  | |  |  |  |  |  | |  |  |
| **Sex** |  |  |  | 0.5 |  |  |  | |  |  | |  |  |  |  |  | | **0.013** |  |
| *Male* | 29,244 (34.2%) | 6,211 (34.3%) | 5,497 (33.7%) |  |  |  | 367 (32.2%) | | 1,216 (36.6%) | 1,171 (33.0%) | | 163 (37.5%) | 269 (33.3%) | 659 (33.2%) | 2,328 (34.1%) | 38 (50.7%) | |  |  |
| *Female* | 56,269 (65.8%) | 11,918 (65.7%) | 10,799 (66.3%) |  |  |  | 772 (67.8%) | | 2,104 (63.4%) | 2,375 (67.0%) | | 272 (62.5%) | 538 (66.7%) | 1,328 (66.8%) | 4,492 (65.9%) | 37 (49.3%) | |  |  |
| **Age at migration (years)** | - | 21 (11, 26) | - |  |  |  | 20 (5, 25) | | 24 (15, 28) | 17 (9, 23) | | 23 (11, 27) | 14 (6, 25) | 22 (15, 27) | 21 (12, 26) | 25 (21, 28) | | **<0.001** |  |
| **Case status** |  |  |  | **<0.001** |  |  |  | |  |  | |  |  |  |  |  | | **<0.001** |  |
| *Control* | 70,484 (82.4%) | 16,703 (92.1%) | 12,809 (78.6%) |  |  |  | 949 (83.3%) | | 3,057 (92.1%) | 3,298 (93.0%) | | 364 (83.7%) | 694 (86.0%) | 1,859 (93.6%) | 6,416 (94.1%) | 66 (88.0%) | |  |  |
| *Case* | 15,029 (17.6%) | 1,426 (7.9%) | 3,487 (21.4%) |  |  |  | 190 (16.7%) | | 263 (7.9%) | 248 (7.0%) | | 71 (16.3%) | 113 (14.0%) | 128 (6.4%) | 404 (5.9%) | 9 (12.0%) | |  |  |
| **Cases only** |  |  |  |  |  |  |  | |  |  | |  |  |  |  |  | |  |  |
| **Sex (% male)** | 5067 (33.7%) | 559 (39.2%) | 1177 (33.8%) | **<0.001** |  |  | 57 (30.0%) | | 103 (39.2%) | 96 (38.7%) | | 30 (42.3%) | 40 (35.4%) | 59 (46.1%) | 169 (41.8%) | 5 (55.6%) | | 0.075 |  |
| **Age at diagnosis (years)** | 25.4  (21.2, 30.7) | 28.8  (24.3, 32.9) | 25.4  (21.2, 30.4) | **<0.001** |  |  | 30.2  (24.5, 33.4) | | 29.8  (24.7, 33.8) | 29.1  (24.1, 32.7) | | 31.0  (26.0, 34.1) | 30.3  (26.4, 33.8) | 27.0  (22.1, 31.0) | 27.1  (24.0, 31.5) | 35.1  (34.8, 37.2) | | **<0.001** |  |
| **Diagnosis (SCZ & BD)** |  |  |  | **<0.001** |  |  |  | |  |  | |  |  |  |  |  | | NA |  |
| *BD diagnosis only* | 14,693 (97.8%) | 1,363 (95.6%) | 3,404 (97.6%) |  |  |  | 184 (96.8%) | | 259 (98.5%) | 229 (92.3%) | | 69 (97.2%) | 111 (98.2%) | 114 (89.1%) | 388 (96.0%) | 9 (100.0%) | |  |  |
| *Had SCZ & BD diagnosis* | 336 (2.2%) | 63 (4.4%) | 83 (2.4%) |  |  |  | 6 (3.2%) | | 4 (1.5%) | 19 (7.7%) | | 2 (2.8%) | 2 (1.8%) | 14 (10.9%) | 16 (4.0%) | 0 (0.0%) | |  |  |
| **BD diagnosis subcategory** |  |  |  | **<0.001** |  |  |  | |  |  | |  |  |  |  |  | | NA |  |
| *ICD9* | 138 (0.9%) | 13 (0.9%) | 24 (0.7%) |  |  |  | 3 (1.6%) | | 5 (1.9%) | 2 (0.8%) | | 0 (0.0%) | 1 (0.9%) | 1 (0.8%) | 1 (0.2%) | 0 (0.0%) | |  |  |
| *ICD10 - other* | 13,386 (87.0%) | 1,212 (83.0%) | 3,106 (87.1%) |  |  |  | 166 (87.4%) | | 222 (84.4%) | 197 (79.4%) | | 61 (85.9%) | 100 (88.5%) | 93 (72.7%) | 334 (82.7%) | 8 (88.9%) | |  |  |
| *ICD10 - with psychosis* | 719 (4.7%) | 121 (8.3%) | 188 (5.3%) |  |  |  | 7 (3.7%) | | 14 (5.3%) | 28 (11.3%) | | 5 (7.0%) | 9 (8.0%) | 24 (18.8%) | 31 (7.7%) | 1 (11.1%) | |  |  |
| *ICD10 - without psychosis* | 1,135 (7.4%) | 115 (7.9%) | 248 (7.0%) |  |  |  | 14 (7.4%) | | 22 (8.4%) | 21 (8.5%) | | 5 (7.0%) | 3 (2.7%) | 10 (7.8%) | 38 (9.4%) | 0 (0.0%) | |  |  |
| **Time to diagnosis (migrants, years)** | - | 15 (9, 21) | - |  |  |  | 18 (9, 27) | | 14 (8, 22) | 14 (11, 18) | | 12 (7, 19) | 19 (12, 25) | 13 (9, 18) | 17 (10, 21) | 7 (6, 12) | | **<0.001** |  |

n (column %); Median (IQR). ^1^ Kruskal-Wallis rank sum test for continuous variables, Pearson's Chi-squared test for categorical variables with all expected cell counts >=55. Oceania region excluded from statistical tests. NA, no statistical test performed due to few observations. Bold indicates p<0.05. Migrant status excludes Swedish-born individuals with missing parental ancestry data (n=704, 0.6%).

Table S3 Sex stratified regression models for each migrant exposure category for SCZ

| **Schizophrenia** | | |  |  |  |  |  | |  | |  |  |  |  | |  | |  |  |  |  | |  |
| --- | --- | --- | --- | --- | --- | --- | --- | --- | --- | --- | --- | --- | --- | --- | --- | --- | --- | --- | --- | --- | --- | --- | --- |
|  |  | | **All** | | | | |  | | **Males** | | | | |  | | **Females** | | | | |  | |
|  | Characteristic | | Cases n (%) | IRR | 95% CI | p-value |  | | Cases n (%) | | IRR | 95% CI | p-value |  | | Cases n (%) | | IRR | 95% CI | p-value | P sex differences | |  |
| Baseline model | Swedish ancestry | | 3,057 (59%) | — | — |  |  | | 2,007 (58%) | | — | — |  |  | | 1,050 (63%) | | — | — |  |  | |  |
|  | First-generation migrants | | 1,036 (20%) | **1.28** | 1.19, 1.39 | <0.001 |  | | 745 (21%) | | **1.46** | 1.33, 1.61 | <0.001 |  | | 291 (17%) | | 0.97 | 0.84, 1.12 | 0.7 | **<0.001** | |  |
|  | Second-generation | | 1,070 (21%) | **2.02** | 1.86, 2.19 | <0.001 |  | | 732 (21%) | | **2.08** | 1.89, 2.29 | <0.001 |  | | 338 (20%) | | **1.90** | 1.65, 2.19 | <0.001 | 0.30 | |  |
|  |  | | n (%) | IRR | 95% CI | p-value |  | | n (%) | | IRR | 95% CI | p-value |  | | n (%) | | IRR | 95% CI | p-value | P(sex) | |  |
| Region | Swedish ancestry | | 3,057 (75%) | — | — |  |  | | 2,007 (73%) | | — | — |  |  | | 1,050 (78%) | | — | — |  |  | |  |
|  | Nordic | | 53 (1.3%) | 1.06 | 0.78, 1.43 | 0.7 |  | | 36 (1.3%) | | 1.15 | 0.80, 1.67 | 0.5 |  | | 17 (1.3%) | | 0.91 | 0.54, 1.54 | 0.7 | 0.47 | |  |
|  | EU28 | | 111 (2.7%) | **0.72** | 0.59, 0.88 | 0.002 |  | | 72 (2.6%) | | **0.73** | 0.57, 0.94 | 0.016 |  | | 39 (2.9%) | | **0.70** | 0.49, 0.98 | 0.039 | 0.82 | |  |
|  | EUR (non-EU28) | | 224 (5.5%) | **1.45** | 1.24, 1.70 | <0.001 |  | | 157 (5.7%) | | **1.63** | 1.35, 1.97 | <0.001 |  | | 67 (5.0%) | | 1.16 | 0.88, 1.53 | 0.3 | **0.047** | |  |
|  | North America | | 20 (0.5%) | 1.06 | 0.65, 1.73 | 0.8 |  | | 13 (0.5%) | | 1.02 | 0.56, 1.86 | >0.9 |  | | 7 (0.5%) | | 1.14 | 0.50, 2.64 | 0.8 | 0.83 | |  |
|  | South America | | 43 (1.1%) | **1.43** | 1.01, 2.03 | 0.042 |  | | 33 (1.2%) | | **1.75** | 1.17, 2.63 | 0.007 |  | | 10 (0.7%) | | 0.88 | 0.45, 1.75 | 0.7 | 0.092 | |  |
|  | Africa | | 221 (5.4%) | **2.55** | 2.15, 3.02 | <0.001 |  | | 170 (6.2%) | | **3.07** | 2.51, 3.74 | <0.001 |  | | 51 (3.8%) | | **1.61** | 1.16, 2.24 | 0.005 | **0.0011** | |  |
|  | Asia | | 362 (8.8%) | **1.18** | 1.05, 1.34 | 0.007 |  | | 262 (9.5%) | | **1.39** | 1.20, 1.60 | <0.001 |  | | 100 (7.5%) | | 0.84 | 0.67, 1.06 | 0.14 | **<0.001** | |  |
|  |  | | n (%) | IRR | 95% CI | p-value |  | | n (%) | | IRR | 95% CI | p-value |  | | n (%) | | IRR | 95% CI | p-value | P(sex) | |  |
| Region of origin –  migration in childhood or adulthood | Swedish ancestry | | 3,057 (75%) | — | — |  |  | | 2,007 (73%) | | — | — |  |  | | 1,050 (78%) | | — | — |  |  | |  |
|  | Childhood | Nordic | 47 (1.1%) | **2.56** | 1.78, 3.69 | <0.001 |  | | 33 (1.2%) | | **2.67** | 1.72, 4.13 | <0.001 |  | | 14 (1.0%) | | **2.37** | 1.23, 4.56 | 0.010 | 0.77 | |  |
|  |  | EU28 | 91 (2.2%) | **2.41** | 1.86, 3.12 | <0.001 |  | | 65 (2.4%) | | **2.51** | 1.84, 3.42 | <0.001 |  | | 26 (1.9%) | | **2.19** | 1.36, 3.54 | 0.001 | 0.64 | |  |
|  |  | EUR (non-EU28) | 160 (3.9%) | **2.68** | 2.19, 3.28 | <0.001 |  | | 114 (4.1%) | | **2.95** | 2.31, 3.76 | <0.001 |  | | 46 (3.4%) | | **2.21** | 1.54, 3.17 | <0.001 | 0.19 | |  |
|  |  | North America | 14 (0.3%) | **2.67** | 1.37, 5.22 | 0.004 |  | | 11 (0.4%) | | **2.97** | 1.37, 6.43 | 0.006 |  | | 3 (0.2%) | | 1.90 | 0.48, 7.47 | 0.4 | 0.58 | |  |
|  |  | South America | 42 (1.0%) | **2.51** | 1.71, 3.68 | <0.001 |  | | 33 (1.2%) | | **2.99** | 1.92, 4.67 | <0.001 |  | | 9 (0.7%) | | 1.53 | 0.70, 3.34 | 0.3 | 0.14 | |  |
|  |  | Africa | 144 (3.5%) | **7.23** | 5.54, 9.43 | <0.001 |  | | 120 (4.4%) | | **8.39** | 6.18, 11.4 | <0.001 |  | | 24 (1.8%) | | **4.33** | 2.45, 7.62 | <0.001 | **0.043** | |  |
|  |  | Asia | 256 (6.3%) | **2.75** | 2.34, 3.23 | <0.001 |  | | 191 (6.9%) | | **3.04** | 2.51, 3.67 | <0.001 |  | | 65 (4.8%) | | **2.14** | 1.57, 2.93 | <0.001 | 0.06 | |  |
|  | Adulthood | Nordic | 6 (0.1%) | **0.19** | 0.08, 0.42 | <0.001 |  | | 3 (0.1%) | | **0.15** | 0.05, 0.49 | 0.001 |  | | 3 (0.2%) | | **0.23** | 0.07, 0.75 | 0.014 | 0.62 | |  |
|  |  | EU28 | 20 (0.5%) | **0.17** | 0.11, 0.27 | <0.001 |  | | 7 (0.3%) | | **0.09** | 0.04, 0.20 | <0.001 |  | | 13 (1.0%) | | **0.30** | 0.17, 0.52 | <0.001 | **0.016** | |  |
|  |  | EUR (non-EU28) | 64 (1.6%) | **0.67** | 0.51, 0.87 | 0.003 |  | | 43 (1.6%) | | 0.74 | 0.53, 1.03 | 0.078 |  | | 21 (1.6%) | | **0.55** | 0.35, 0.88 | 0.012 | 0.31 | |  |
|  |  | North America | 6 (0.1%) | 0.45 | 0.19, 1.03 | 0.059 |  | | 2 (<0.1%) | | **0.22** | 0.05, 0.92 | 0.038 |  | | 4 (0.3%) | | 0.90 | 0.31, 2.67 | 0.9 | 0.12 | |  |
|  |  | South America | 1 (<0.1%) | **0.07** | 0.01, 0.51 | 0.009 |  | | 0 (0%) | | 0.00 | 0.00, Inf | >0.9 |  | | 1 (<0.1%) | | 0.17 | 0.02, 1.28 | 0.085 | 0.99 | |  |
|  |  | Africa | 77 (1.9%) | 1.11 | 0.86, 1.43 | 0.4 |  | | 50 (1.8%) | | 1.15 | 0.84, 1.58 | 0.4 |  | | 27 (2.0%) | | 1.02 | 0.67, 1.57 | >0.9 | 0.66 | |  |
|  |  | Asia | 106 (2.6%) | **0.50** | 0.41, 0.61 | <0.001 |  | | 71 (2.6%) | | **0.57** | 0.44, 0.73 | <0.001 |  | | 35 (2.6%) | | **0.40** | 0.28, 0.57 | <0.001 | 0.12 | |  |

|  | |  | n (%) | IRR | 95% CI | p-value |  | n (%) | IRR | 95% CI | p-value |  | n (%) | IRR | 95% CI | p-value | P(sex) |
| --- | --- | --- | --- | --- | --- | --- | --- | --- | --- | --- | --- | --- | --- | --- | --- | --- | --- |
| Migrant  status | Swedish ancestry | | 3,114 (60%) | — | — |  |  | 2,048 (58%) | — | — |  |  | 1,066 (63%) | — | — |  |  |
|  | Child migrants | | 785 (15%) | **2.92** | 2.66, 3.22 | <0.001 |  | 588 (17%) | **3.20** | 2.86, 3.58 | <0.001 |  | 197 (12%) | **2.33** | 1.95, 2.79 | <0.001 | **0.004** |
|  | Adult migrants | | 251 (4.8%) | **0.45** | 0.39, 0.51 | <0.001 |  | 157 (4.5%) | **0.46** | 0.39, 0.55 | <0.001 |  | 94 (5.5%) | **0.43** | 0.34, 0.53 | <0.001 | 0.60 |
|  | Second-generation with 1 migrant parent | | 587 (11%) | **1.72** | 1.55, 1.90 | <0.001 |  | 400 (11%) | **1.77** | 1.56, 2.00 | <0.001 |  | 187 (11%) | **1.62** | 1.36, 1.94 | <0.001 | 0.44 |
|  | Second-generation with 2 migrant parents | | 483 (9.3%) | **2.48** | 2.21, 2.78 | <0.001 |  | 332 (9.4%) | **2.54** | 2.21, 2.92 | <0.001 |  | 151 (8.9%) | **2.36** | 1.93, 2.90 | <0.001 | 0.57 |
| Migrant parents | Only mother  Swedish-born father) | | 270 (6.5%) | **1.47** | 1.27, 1.70 | <0.001 |  | 183 (6.6%) | **1.45** | 1.21, 1.72 | <0.001 |  | 87 (6.2% | **1.52** | 1.18, 1.96 | 0.001 | 0.70 |
|  | Only father  (Swedish-born mother) | | 317 (7.7%) | **1.73** | 1.51, 1.98 | <0.001 |  | 217 (7.9%) | **1.78** | 1.51, 2.11 | <0.001 |  | 100 (7.2%) | **1.62** | 1.28, 2.05 | <0.001 | 0.50 |

IRR = Incidence rate ratio, CI = Confidence Interval; P(sex), differences in IRR between sexes evaluated using Wald-tests. n (%) corresponds to number of cases. Bold indicates p<0.05. Regression model estimates are relative to Swedish ancestry. Aside from the baseline model, children of migrants are excluded. Child <18 years old; Adult≥18 years old.

Table S4 Sex stratified regression models for each migrant exposure category for BD

| **Bipolar disorder** | | |  |  |  |  |  |  |  |  |  |  |  |  |  | | |
| --- | --- | --- | --- | --- | --- | --- | --- | --- | --- | --- | --- | --- | --- | --- | --- | --- | --- |
|  |  | | **All** | | | | **Males** | | | | **Females** | | | | |  |  |
|  | Characteristic | | n (%) | IRR | 95% CI | p-value | n (%) | IRR | 95% CI | p-value | n (%) | IRR | 95% CI | p-value | P sex differences | | |
| Baseline model | Swedish ancestry | | 15,029 (75.4%) | — | — |  | 5,067 (74.4%) | — | — |  | 9,962 (75.8%) | — | — |  |  | | |
|  | First-generation | | 1,426 (7.2%) | **0.40** | 0.37, 0.42 | <0.001 | 559 (8.2%) | **0.47** | 0.43, 0.51 | <0.001 | 867 (6.6%) | **0.36** | 0.33, 0.39 | <0.001 | **<0.001** | | |
|  | Second-generation | | 3,487 (17.5%) | **1.28** | 1.23, 1.34 | <0.001 | 1,177 (17.3%) | **1.30** | 1.21, 1.40 | <0.001 | 2,310 (17.6%) | **1.28** | 1.21, 1.34 | <0.001 | 0.65 | | |
|  |  | | n (%) | IRR | 95% CI | p-value | n (%) | IRR | 95% CI | p-value | n (%) | IRR | 95% CI | p-value | P(sex) | | |
| Region | Swedish ancestry | | 15,029 (91%) | — | — |  | 5,067 (90%) | — | — |  | 9,962 (92%) | — | — |  |  | | |
|  | Nordic | | 190 (1.2%) | 0.91 | 0.78, 1.07 | 0.3 | 57 (1.0%) | 0.83 | 0.62, 1.12 | 0.2 | 133 (1.2%) | 0.95 | 0.78, 1.15 | 0.6 | 0.46 | | |
|  | EU28 | | 263 (1.6%) | **0.40** | 0.35, 0.45 | <0.001 | 103 (1.8%) | **0.44** | 0.35, 0.54 | <0.001 | 160 (1.5%) | **0.37** | 0.32, 0.44 | <0.001 | 0.25 | | |
|  | EUR (non-EU28) | | 248 (1.5%) | **0.35** | 0.30, 0.39 | <0.001 | 96 (1.7%) | **0.41** | 0.33, 0.51 | <0.001 | 152 (1.4%) | **0.31** | 0.27, 0.37 | <0.001 | 0.055 | | |
|  | North America | | 71 (0.4%) | 0.90 | 0.70, 1.17 | 0.4 | 30 (0.5%) | 1.04 | 0.69, 1.56 | 0.9 | 41 (0.4%) | 0.83 | 0.59, 1.16 | 0.3 | 0.40 | | |
|  | South America | | 113 (0.7%) | **0.76** | 0.62, 0.94 | 0.009 | 40 (0.7%) | 0.80 | 0.56, 1.13 | 0.2 | 73 (0.7%) | **0.75** | 0.58, 0.96 | 0.023 | 0.77 | | |
|  | Africa | | 128 (0.8%) | **0.32** | 0.27, 0.38 | <0.001 | 59 (1.0%) | **0.48** | 0.36, 0.63 | <0.001 | 69 (0.6%) | **0.25** | 0.19, 0.32 | <0.001 | **<0.001** | | |
|  | Asia | | 404 (2.5%) | **0.29** | 0.26, 0.32 | <0.001 | 169 (3.0%) | **0.37** | 0.31, 0.43 | <0.001 | 235 (2.2%) | **0.26** | 0.22, 0.29 | <0.001 | **<0.001** | | |
|  |  | | n (%) | IRR | 95% CI | p-value | n (%) | IRR | 95% CI | p-value | n (%) | IRR | 95% CI | p-value | P(sex) | | |
| Region of origin - migration in childhood or adulthood | Swedish ancestry | | 15,029 (91%) | — | — |  | 5,067 (90%) | — | — |  | 9,962 (92%) | — | — |  |  | | |
|  | Childhood | Nordic | 131 (0.8%) | **1.59** | 1.29, 1.95 | <0.001 | 44 (0.8%) | **1.60** | 1.12, 2.28 | 0.010 | 87 (0.8%) | **1.59** | 1.23, 2.04 | <0.001 | 0.98 | | |
|  |  | EU28 | 167 (1.0%) | 1.02 | 0.86, 1.21 | 0.8 | 63 (1.1%) | 1.12 | 0.84, 1.49 | 0.4 | 104 (1.0%) | 0.96 | 0.77, 1.20 | 0.7 | 0.41 | | |
|  |  | EUR (non-EU28) | 165 (1.0%) | **0.46** | 0.39, 0.54 | <0.001 | 63 (1.1%) | **0.54** | 0.41, 0.71 | <0.001 | 102 (0.9%) | **0.43** | 0.35, 0.52 | <0.001 | 0.17 | | |
|  |  | North America | 34 (0.2%) | 1.20 | 0.82, 1.77 | 0.3 | 15 (0.3%) | 1.51 | 0.83, 2.76 | 0.2 | 19 (0.2%) | 1.04 | 0.63, 1.73 | 0.9 | 0.36 | | |
|  |  | South America | 85 (0.5%) | 1.05 | 0.82, 1.33 | 0.7 | 32 (0.6%) | 1.06 | 0.71, 1.57 | 0.8 | 53 (0.5%) | 1.04 | 0.77, 1.41 | 0.8 | 0.95 | | |
|  |  | Africa | 94 (0.6%) | 0.81 | 0.65, 1.01 | 0.067 | 45 (0.8%) | 1.20 | 0.86, 1.69 | 0.3 | 49 (0.5%) | **0.62** | 0.46, 0.84 | 0.002 | **0.005** | | |
|  |  | Asia | 309 (1.9%) | **0.61** | 0.54, 0.69 | <0.001 | 131 (2.3%) | **0.73** | 0.60, 0.88 | 0.001 | 178 (1.6%) | **0.55** | 0.47, 0.64 | <0.001 | **0.022** | | |
|  | Adulthood | Nordic | 59 (0.4%) | **0.46** | 0.35, 0.60 | <0.001 | 13 (0.2%) | **0.31** | 0.18, 0.55 | <0.001 | 46 (0.4%) | **0.53** | 0.39, 0.72 | <0.001 | 0.10 | | |
|  |  | EU28 | 96 (0.6%) | **0.19** | 0.15, 0.23 | <0.001 | 40 (0.7%) | **0.22** | 0.16, 0.30 | <0.001 | 56 (0.5%) | **0.17** | 0.13, 0.22 | <0.001 | 0.26 | | |
|  |  | EUR (non-EU28) | 83 (0.5%) | **0.23** | 0.18, 0.28 | <0.001 | 33 (0.6%) | **0.28** | 0.19, 0.39 | <0.001 | 50 (0.5%) | **0.20** | 0.15, 0.27 | <0.001 | 0.19 | | |
|  |  | North  America | 37 (0.2%) | 0.73 | 0.51, 1.04 | 0.081 | 15 (0.3%) | 0.78 | 0.45, 1.36 | 0.4 | 22 (0.2%) | 0.70 | 0.44, 1.11 | 0.13 | 0.77 | | |
|  |  | South  America | 28 (0.2%) | **0.41** | 0.27, 0.60 | <0.001 | 8 (0.1%) | **0.39** | 0.19, 0.80 | 0.011 | 20 (0.2%) | **0.42** | 0.26, 0.66 | <0.001 | 0.87 | | |
|  |  | Africa | 34 (0.2%) | **0.12** | 0.08, 0.17 | <0.001 | 14 (0.2%) | **0.16** | 0.09, 0.27 | <0.001 | 20 (0.2%) | **0.10** | 0.06, 0.16 | <0.001 | 0.19 | | |
|  |  | Asia | 95 (0.6%) | **0.11** | 0.09, 0.13 | <0.001 | 38 (0.7%) | **0.13** | 0.10, 0.19 | <0.001 | 57 (0.5%) | **0.09** | 0.07, 0.12 | <0.001 | 0.11 | | |

|  |  | n (%) | IRR | 95% CI | p-value | n (%) | IRR | 95% CI | p-value | n (%) | IRR | 95% CI | p-value | P(sex) |
| --- | --- | --- | --- | --- | --- | --- | --- | --- | --- | --- | --- | --- | --- | --- |
| Migrant status | Swedish ancestry | 15,194 (76%) | — | — |  | 5,129 (75%) | — | — |  | 10,065 (76%) | — | — |  |  |
|  | Child migrants | 1,001 (5.0%) | **0.73** | 0.68, 0.78 | <0.001 | 400 (5.8%) | **0.87** | 0.78, 0.97 | 0.012 | 601 (4.5%) | **0.66** | 0.60, 0.72 | <0.001 | **<0.001** |
|  | Adult migrants | 425 (2.1%) | **0.19** | 0.17, 0.21 | <0.001 | 159 (2.3%) | **0.21** | 0.18, 0.25 | <0.001 | 266 (2.0%) | **0.18** | 0.15, 0.20 | <0.001 | 0.061 |
|  | Second-generation with 1 migrant parent | 2,503 (12%) | **1.45** | 1.38, 1.52 | <0.001 | 822 (12%) | **1.44** | 1.32, 1.57 | <0.001 | 1,681 (13%) | **1.46** | 1.38, 1.55 | <0.001 | 0.80 |
|  | Second-generation with 2 migrant parents | 984 (4.9%) | 0.99 | 0.92, 1.06 | 0.8 | 355 (5.2%) | 1.06 | 0.94, 1.20 | 0.3 | 629 (4.8%) | 0.95 | 0.87, 1.04 | 0.3 | 0.15 |
| Migrant parents | Only mother  (Swedish-born father) | 1,097 (5.9%) | 1.343 | 1.25,1.44 | <0.001 | 368 (5.9%) | 1.384 | 1.23,1,56 | <0.001 | 729 (5.9%) | 1.324 | 1.24,1.44 | <0.001 | 0.20 |
|  | Only father  (Swedish-born mother) | 1,406 (7.6%) | 1.562 | 1.47,1.67 | <0.001 | 454 (7.3%) | 1.504 | 1.35,1.68 | <0.001 | 952 (7.7%) | 1.591 | 1.47,1.72 | <0.001 | 0.08 |

IRR = Incidence rate ratio, CI = Confidence Interval; P(sex), differences in IRR between sexes evaluated using Wald-tests. n (%) corresponds to number of cases. Bold indicates p<0.05. Regression model estimates are relative to Swedish ancestry. Aside from the baseline model, children of migrants are excluded. Child <18 years old; Adult≥18 years old.

# Sensitivity analysis – without buffer period

Figure 1 disease risk as smooth function (spline) of age at migration with and without any imposed (4 year) buffer period.

The reference group is Swedish ancestry. Points or solid lines represent IRR, and bars or shaded areas the corresponding 95% confidence intervals.
Without a buffer period, the number of first-generation migrant SCZ cases increased from 1036 to 1355, and migrant BD cases from 1426 to 1896. Patterns of risk were strikingly similar, however, imposing the buffer criteria leads to minor shifts in the risk curve for SCZ, while BD remains largely unchanged bar the exclusion of the flared confidence intervals on the right hand side tail.
